# 

**Published:** 1992-12

**Authors:** 


W1 WE403

V.107 NO.3 1992

C.01--------SEQ: SR0068788

TI: WEST OF ENGLAND MEDICAL

JOURNAL

### WEST OF ENGLAND MEDICAL JOURNAL

Volume 107(iii) December 1992

NATIONAL LIBRARY
JUN 11 1993
OF MEDICINE

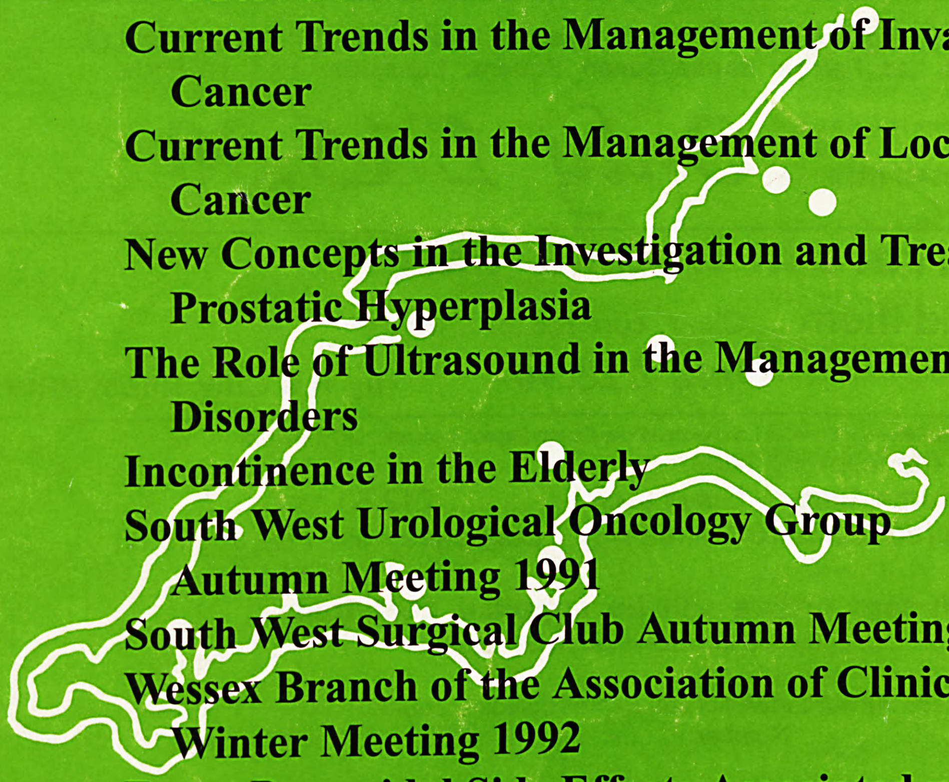

**Urology Mini Symposium**
**Guest Editor — Dr. J. Kabala**
**Current Trends in the Management of Invasive Bladder Cancer**
**Current Trends in the Management of Localised Prostate Cancer**
**New Concepts in the Investigation and Treatment of Prostatic Hyperplasia**
**The Role of Ultrasound in the Management of Scrotal Disorders**
**Incontinence in the Elderly**
**South West Urological Oncology Group Autumn Meeting 1991**
**South West Surgical Club Autumn Meeting 1992**
**Wessex Branch of the Association of Clinical Pathologists Winter Meeting 1992**
**Extra-Paramidal Side Effects Associated with Paroxetine**
**Neuroborreliosis in South Wales**
**Occupational Therapy — Does the Doctor Know?**
**Obituaries**

FORMERLY BRISTOL MEDICO-CHIRURGICAL JOURNAL

ISSN: 0960 6440 NLM Code B5Q

